# Development and validation of a prognostic model for predicting survival and immunotherapy benefits in melanoma based on metabolism-relevant genes

**DOI:** 10.1007/s12672-025-03186-8

**Published:** 2025-07-12

**Authors:** Xiaoru Pan, Shiyao Zhou, Ning Mao, Fengming Yao, Yajun Guo, Wenming Zhou, Shengxiu Liu

**Affiliations:** 1 Department of Dermatology, The First Affiliated Hospital of Anhui Medical University, 218 Jixi Road, Shushan District, Hefei, 230022 Anhui China; 2 Key Laboratory of Dermatology, Anhui Medical University, Ministry of Education, Hefei, 230022 Anhui China; 3 Inflammation and Immune-Mediated Diseases Laboratory of Anhui Province, Hefei, 230022 Anhui China; 4 Department of Dermatology, Anqing Medical Center of Anhui Medical University, Anqing, 246000 Anhui China

**Keywords:** Cutaneous melanoma, Metabolism-related genes (MRGs), Prognosis, Immunotherapy, Pathology features, Single-cell, Machine learning

## Abstract

Skin cutaneous melanoma (SKCM) is a fatal form of skin cancer. Metabolism-related genes (MRGs) comprise a group of genes that possess the ability to modulate and regulate metabolic pathways. In this study, the expression levels of MRGs were used to classify SKCM patients into three molecular subtypes. Then the differentially expressed genes (DEGs) among the three MRGs molecular clusters were applied into the LASSO and COX regression analysis to identify five signature genes. Furthermore, we developed a prognostic model to predict the prognosis of SKCM patients and evaluate the response of SKCM patients to immunotherapy based on the expression of signature genes. Pathological features were extracted using the ResNet50 deep learning framework and Cellprofiler software, and feature selection was performed using elastic regression and univariate Cox analysis to obtain 13 pathological features related to the MRGs prognostic model. Single-cell RNA sequencing (scRNA-seq) analysis identified the expression of MRGs in multiple cell types and found that SLC5A3 + malignant cells may mediate potential communication with tumor-associated fibroblasts through the PI3K-AKT pathway and cholesterol metabolism pathway. It is worth noting that, through in vitro experiments, including western blot (WB), quantitative PCR (qPCR), and immunohistochemistry (IHC) techniques, we found differences in the expression levels of signature genes between normal melanocytes and SKCM cells. In addition, suppressing the expression of the signature gene, SLC5A3, in a SKCM cell line through the utilization of small interfering RNAs (siRNAs) could inhibit the proliferation and migration of cells, as evidenced by the implementation of colony formation assay, CCK8, and cell transfection techniques.

**Supplementary Information:**

The online version contains supplementary material available at 10.1007/s12672-025-03186-8.

## Introduction

Skin cutaneous melanoma (SKCM) is a highly fatal kind of skin cancer that can manifest in various regions of the body because of the malignant conversion of melanocytes [1]. Globally, SKCM accounts for approximately 0.7% of overall cancer-related deaths [2]. Furthermore, the incidence and mortality rates of SKCM exhibit variations among different countries, with higher rates observed in Australia and European nations, and lower rates in Africa. These disparities are attributed to various factors influencing the development of the disease, such as ethnicity, lifestyle, and genetic background [3]. The pathogenesis of melanoma is intricate, involving intricate interactions between intrinsic tumor factors and surrounding immune factors [4]. Despite the widespread use and significant progress of current targeted therapy and immunotherapy in the treatment of melanoma, the prognosis of patients remains unfavorable [5]. Hence, it is imperative to investigate the prognostic factors of SKCM patients.

Metabolic reprogramming is a key feature in cancer biology, particularly in the development of malignant tumors. The Warburg effect, where cancer cells prefer glycolysis over oxidative phosphorylation for energy under aerobic conditions, has been observed in various types of cancer [6]. In melanoma, metabolic reprogramming also plays a key role. Research shows that melanoma cells use metabolic reprogramming to support the acquisition and maintenance of their invasive phenotype. Specifically, the S100A4 protein promotes the migration and invasion of melanoma cells while regulating the expression of metabolic genes, thereby influencing the inhibition of mitochondrial respiration and the activation of glycolytic flux [7]. Recent studies have shown that melanoma cells exhibit significant metabolic plasticity. Melanoma cells can produce adenosine triphosphate (ATP) through both cytoplasmic and mitochondrial pathways, but their energy requirements primarily depend on glycolysis. This metabolic flexibility not only supports melanoma growth and progression but also promotes the development of resistance to BRAF/MEK inhibitors [8]. In the progression of melanoma, abnormalities in lipid and cholesterol metabolism are also considered significant factors [9]. Research has shown that after melanoma cells develop resistance to BRAF inhibitors, they become more dependent on lipid metabolism [10]. Furthermore, drug combinations targeting lipid metabolism, such as inhibiting cholesterol acetylation (ACAT/SOAT), can enhance the sensitivity of melanoma cells to BRAF inhibitors by inducing ferroptosis [11].

Numerus signature genes have now been discovered for forecasting the outcomes of cancer, and they will be combined with immunotherapy databases to validate. Those studies also incorporate data related to tumor mutation burden (TMB), tumor microenvironment (TME), and so on to evaluate prognostic models [12, 13]. To date, there is a lack of established prognostic models for SKCM that are based on metabolism-related genes (MRGs).

This study involved the collection and utilization of 2752 previously published MRGs encoding all known human metabolic enzymes and transporters to identify five predictive signature genes, namely FCGR2C, GBP4, CHRDL1, SLC5A3, and GJA1.

Then we established a prognostic model for predicting the prognosis of SKCM based on the signature genes. It also has the potential to evaluate the response of patients with varying risk levels to immunotherapy. In addition, we obtained multiple single-cell datasets to identify the expression levels of MRGs in different cell types. Interestingly, SLC5A3 was mainly expressed in malignant melanoma cells. Furthermore, we used the CommPath software package to identify the communication between SLC5A3 + and other cell subpopulations in the microenvironment, and proposed receptor-ligand interactions that may mediate potential communication. The interaction of these receptor-ligands may also mediate the activation of metabolic pathways such as PI3K-AKT. Furthermore, our findings indicate that the expression of a signature gene, SLC5A3, contributes to the migration and proliferation of SKCM cells.

## Results

### Genetic alterations of MRGs in SKCM patients

**Fig. 1:**
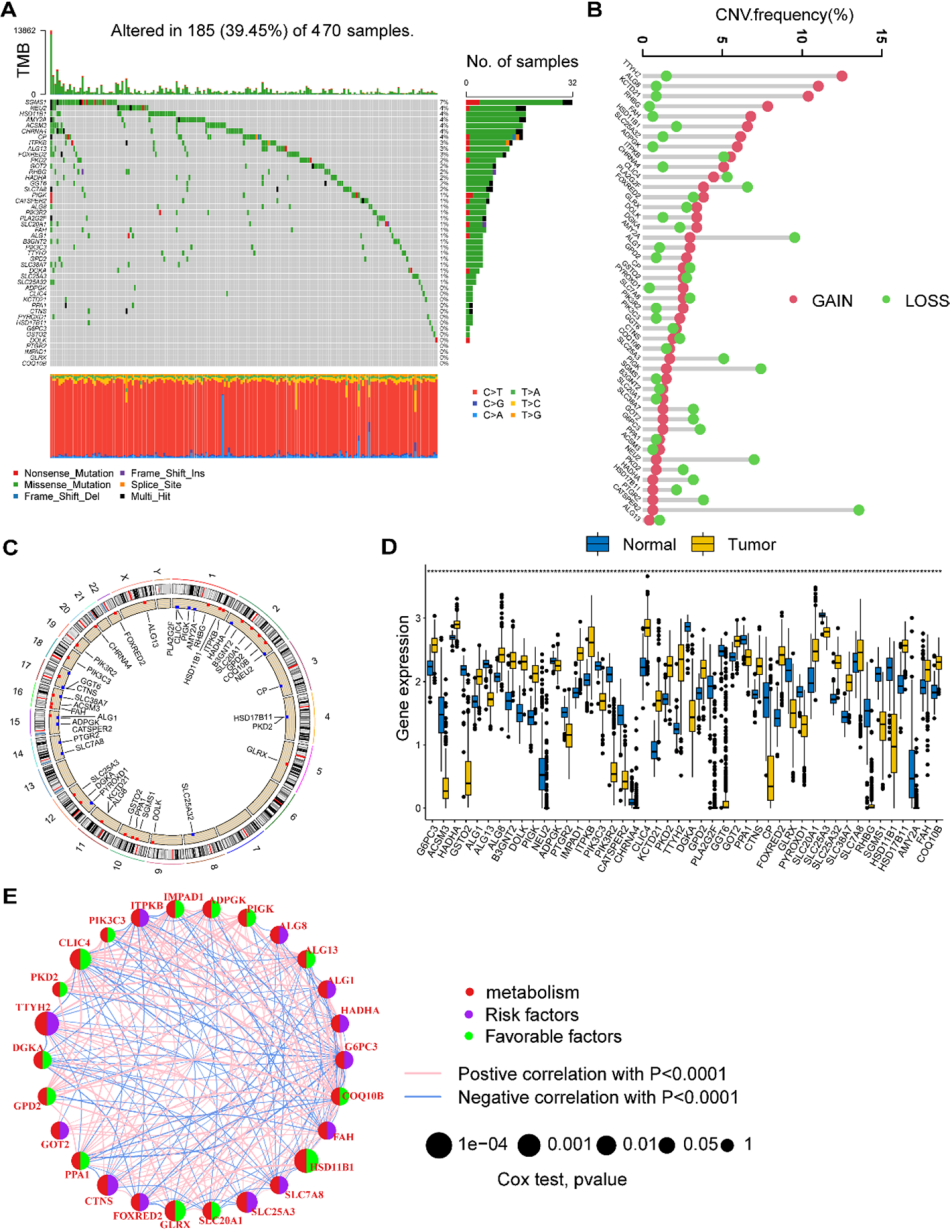
The genetic alteration of MRGs in SKCM. **A** A landscape of somatic mutation rates of MRGs in SKCM patients. **B** Gains and losses of CNV among MRGs. **C** The locations of CNV alterations of MRGs on 23 chromosomes. **D** Differentially expressed MRGs between SKCM patients and normal controls. **E** Interactions among MRGs in SKCM. Blue and red lines represent positive and negative correlations respectively among the genes. *p < 0.05; **p < 0.01; ***p < 0.001

To examine the genetic modification of MRGs in SKCM patients, we conducted an analysis on the frequency of mutations and copy number variations (CNVs) for the genes. We collected the mutation data, the CNV data, and the transcriptional data of 470 SKCM patients from TCGA database. Among the SKCM patients, a total of 185 patients exhibited mutations in MRGs, resulting in a mutational frequency of 39% (Fig. 1A). We conducted a comprehensive investigation into the CNVs observed among the MRGs in patients with SKCM. Our findings revealed that TTYH2, ALG8, KCTD21, RHBG, FAH, and HSD11B1 exhibited an increase in CNV, whereas AMY2A, SLC25A3, PIGK, NEU2, and CATSPER2 displayed a decrease in CNV (Fig. 1B). Additionally, we provided a detailed depiction of the chromosomal locations where CNVs occur among the MRGs in the human genome (Fig. 1C). Our investigation also revealed the differentially expressed MRGs between individuals diagnosed with SKCM and those in a control group (Fig. 1D). The transcriptional data of 838 healthy controls were collected from the GTEx database. Furthermore, Fig. 1E visually depicts the interaction between prognostically significant MRGs and their p-values of prognostic significance in SKCM patients. Based on the analyses, it was ascertained that SKCM patients exhibited alterations in MRGs.

### Consensus clustering identifying three MRGs clusters

**Fig. 2:**
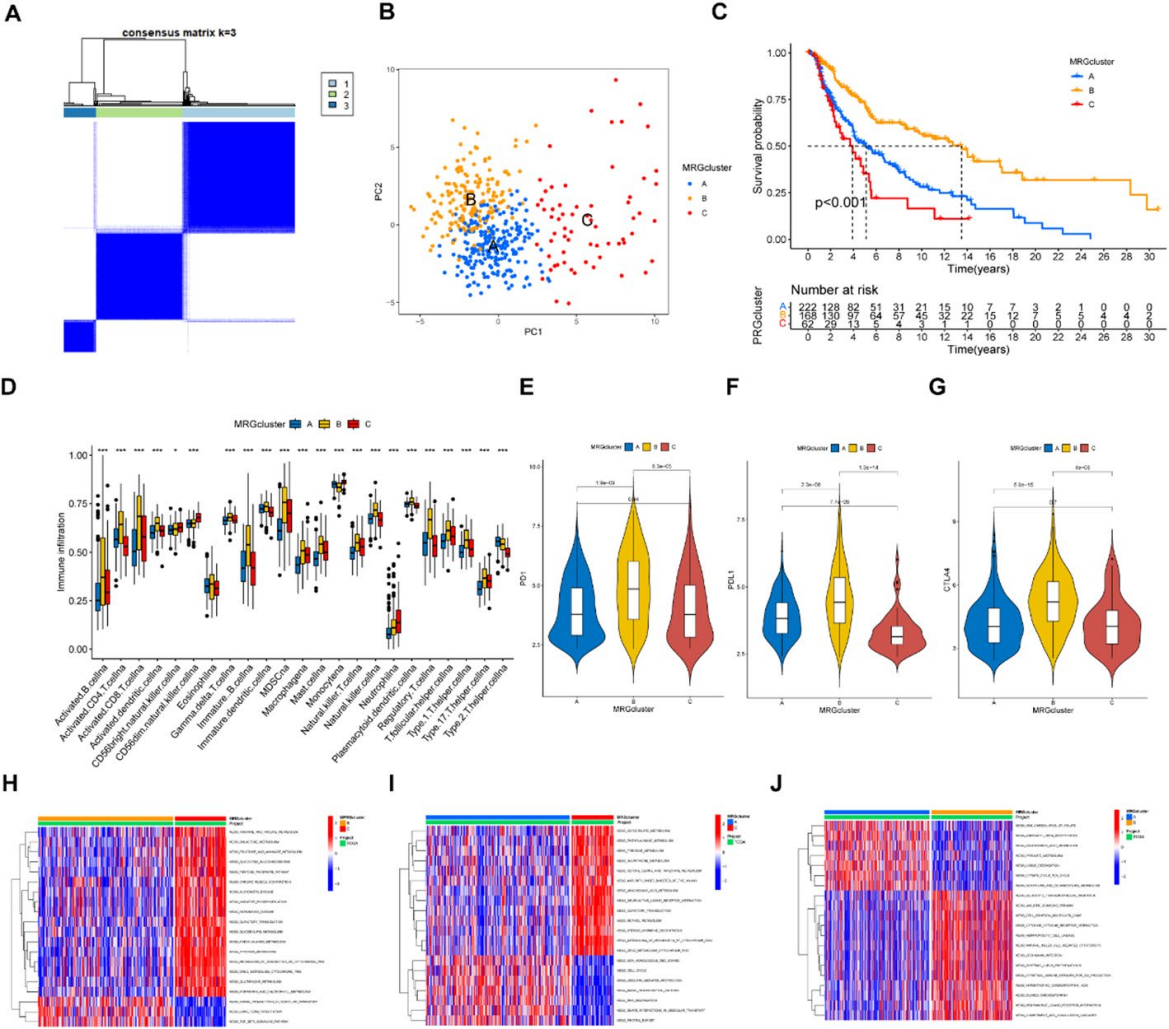
The comparison of clinical and immune related characteristics among the three MRGs molecular clusters. **A** The consensus clustering analysis identified three MRG clusters. **B** PCA revealed three MRGs clusters. **C** KM curves showed significant differences among the three MRGs clusters in survival time (*p* < 0.001). **D** Single-sample gene set enrichment analysis (ssGSEA) showed the significant differences of immune infiltration among the three MRGs clusters. **E**–**G** The expression levels of immune checkpoint genes were different among the three MRGs clusters. **H**–**J** Each two MRG clusters were shown to have enriched pathways using GSVA Gene Set Variation Analysis (GSVA). *p < 0.05; **p < 0.01; ***p < 0.001

The 470 SKCM patients were divided into three MRGs clusters (MRGs cluster A, B and C) by consensus clustering according to MRGs expression levels (Fig. 2A). The PCA showed the individuals were categorized into three groups (Fig. 2B). The Kaplan-Meier (K-M) curve demonstrated that MRGs cluster B had a higher overall survival (OS) compared to clusters A and C (Fig. 2C, *P* < 0.001). These results suggested that the expression of MRGs can affect the survival of SKCM patients. Furthermore, according to the single-sample Gene Set Enrichment Analysis (ssGSEA) results, molecular cluster B exhibited greater levels of immune cell infiltration compared to clusters A and C (Fig. 2D, *p* < 0.05). The molecular cluster B also exhibited higher expression levels of immune checkpoint.

genes, such as PD-1, PD-L1, and CTLA-4, compared to clusters A and C (Fig. 2E-G, *p* < 0.05). Additionally, there were many different enriched pathways between each of the two MRGs molecular clusters (Fig. 2H–J). The higher expression levels of immune checkpoint genes implied that patients would have better clinical efficacy of immune checkpoint inhibitors (ICIs), which may improve the survival of SKCM patients [14].

### Consensus clustering identified gene clusters

**Fig. 3:**
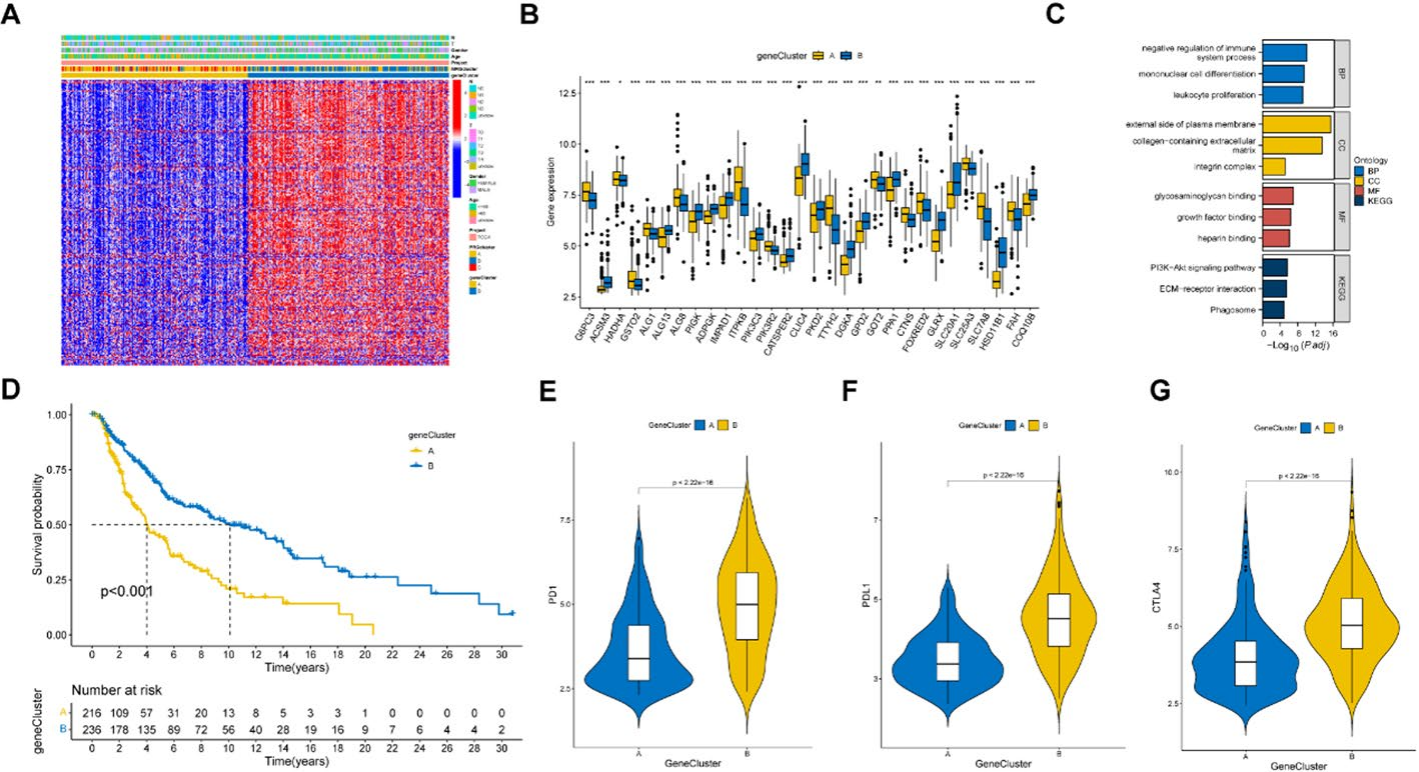
The comparison of clinical and immune related characteristics among the two gene clusters. **A** A Heatmap showed the clinical features and MRGs expressions of the two gene clusters. **B** Differentially expressed MRGs between the two gene clusters. **C** GO and KEGG analysis of the differentially expressed genes (DEGs) among the three molecular clusters. **D** KM curves showed significant differences between the two gene clusters in survival time (*p* < 0.001). **E**–**G** The expression levels of immune checkpoint genes were different among the two gene clusters. *p < 0.05; **p < 0.01; ***p < 0.001

We further identified DEGs among the three MRGs clusters and divided 470 SKCM patients into two clusters (gene clusters A and B) based on these DEGs. The clinical characteristics and DEG expression levels of the two gene clusters were presented in a heatmap (Fig. 3A). The expression levels of many MRGs were different between the two gene subtypes (Fig. 3B). These DEGs were primarily correlated with the BP of regulation of the immune system process, the CC of the external side of the plasma membrane, and the MF of glycosaminoglycan binding, and participated in the PI3K-Akt signaling pathways (Fig. 3C). The K-M curve demonstrated that gene cluster B had a higher OS compared to cluster A (Fig. 3D, *p* < 0.001). In addition, the immune checkpoint genes of cluster B were also more highly expressed than those of cluster A (Fig. 3E-G). These results suggested that DEGs identified according to MRGs clusters were linked with the survival of patients with SKCM.

### Construction and validation of prognostic signature

**Fig. 4:**
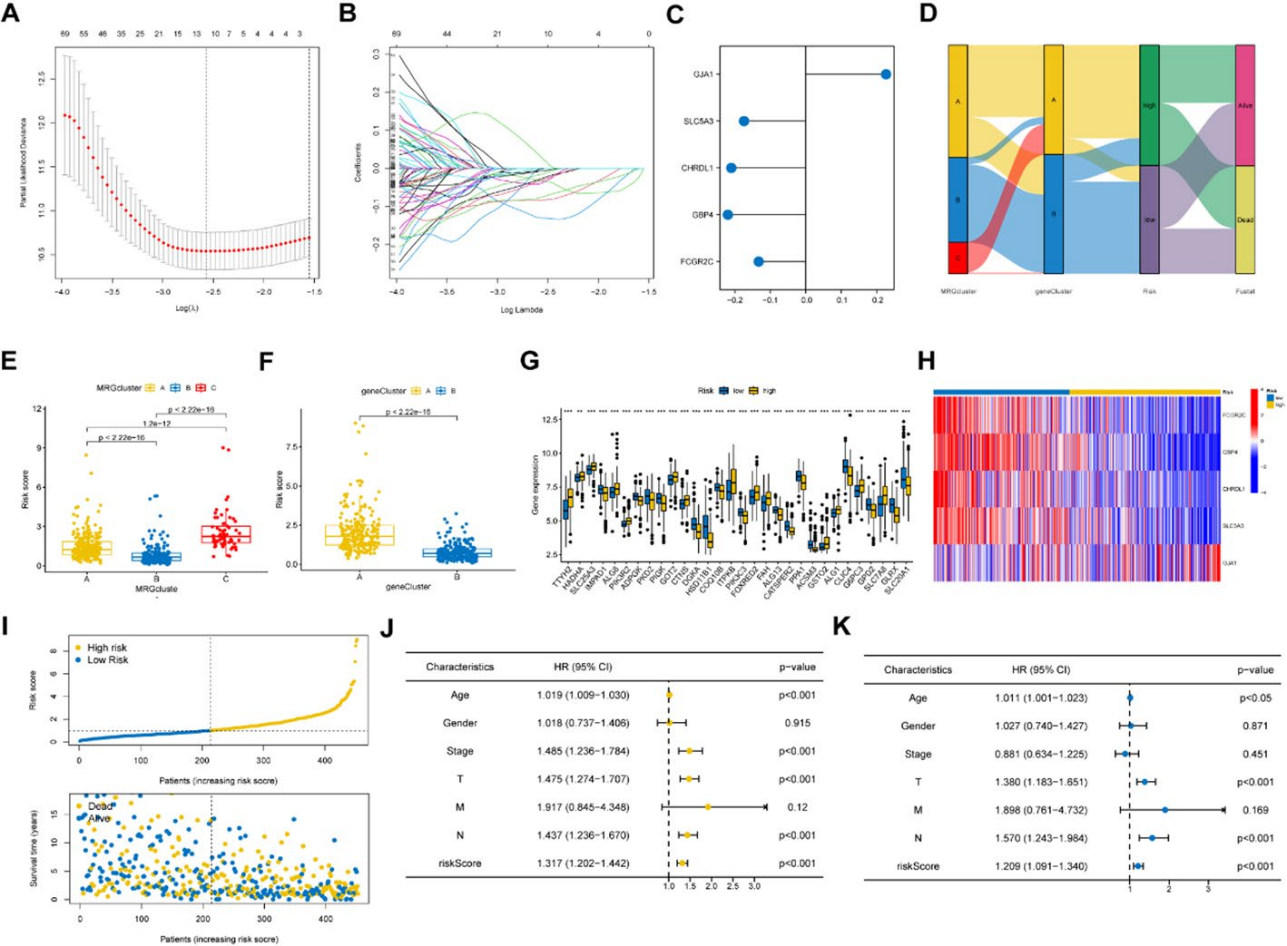
Establishment of the prognostic signature model. **A**, **B** LASSO regression analysis and partial likelihood deviance on the prognostic genes. **C** The risk coefficient of the five signature genes were identified by cox regression analysis. **D** A sankey diagram shows three classifications of the SKCM patients based on MRGs expression and DEGs gene expression, as well as risk scores. **E**, **F** The MRGs molecular cluster B and gene cluster B both had lower risk scores than other clusters (*p* < 0.001). **G** There are significant differences in the expression of many MRGs between the high- and low-risk groups. **H** A heatmap demonstrated FCGR2C, GBP4, CHRDL1 and SLC5A3 had higher expression in the low-risk group, while GJA1 had a lower expression. **I** The high-risk group also has a higher mortality rate.(**J**, **K** Univariate and multivariate cox regression revealing that the risk coefficient of age, gender, tumor staging, and risk score for the SKCM patients. *p < 0.05; **p < 0.01; ***p < 0.001

We identified 5 signature genes, GJA1, SLC5A3, CHRDL1, GBP4, and FCGR2C, with prognostic efficacy from DEGs between the three MRGs clusters by using lasso and Cox regression analysis and their risk coefficient values (Fig. 4A&B), and their risk coefficient values were shown in Fig. 4C. The risk score was determined according to the expression levels of signature genes and their risk coefficient values (Supplementary Table S1) using the formula: [expression level of *GJA1* × (0.2266645)] + [expression level of *SLC5A3* × (-0.1741526)] + [expression level of *CHRDL1* × (-0.2099985)] + [expression level of *GBP4* × (-0.2197066)] + [expression level of *FCGR2C* × (-0.1327172)]. Based on the median risk score, 470 SKCM patients could be divided into high- and low-risk groups. The Sankey diagram showed several classifications of the SKCM patients based on MRG expression and DEG expression, as well as risk scores (Fig. 4D). Both molecular cluster B and gene cluster B, which had better survival rates, exhibited lower risk scores (Fig. 4E&F). Significant differences in the expression of numerous MRGs were observed between the high- and low-risk groups (Fig. 4G). A heatmap demonstrated that FCGR2C, GBP4, CHRDL1, and SLC5A3 had higher expression in the low-risk group, while GJA1 had a lower expression (Fig. 4H). In addition, it can be observed that the group at greater risk also experiences an elevated mortality rate (Fig. 4I). Univariate (Fig. 4J) and multivariate (Fig. 4K) Cox regression indicated that age, sex, tumor stage, and risk score could be independent predictive factors.

**Fig. 5:**
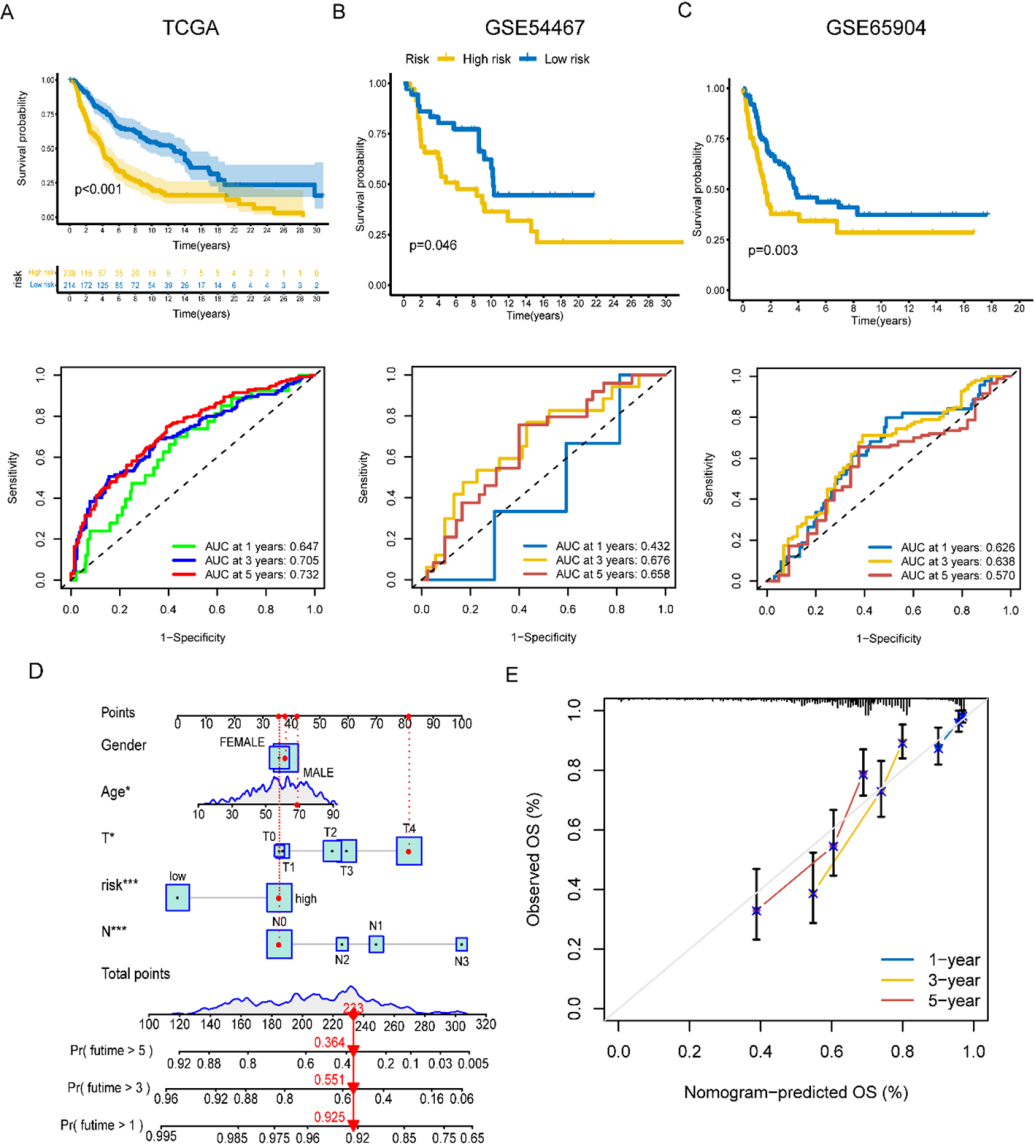
Predicting survival of tumor patients based on risk scores and comprehensive scores. **A**–**C** K-M curves and ROC curves showed the prognostic value in training and validation cohorts. **D** A nomogram shows comprehensive scores to predict the prognosis of SKCM patients. **E** The calibration plots demonstrate the prognostic accuracy of the nomogram

K-M curves and area under curves (AUC) were utilized to evaluate the precision of risk score in predicting the survival of SKCM patients. The K-M curves for training cohort from TCGA showed that low-risk patients had a longer OS (*p* < 0.001), with the 1-, 3-, and 5-year AUC values being 0.647, 0.705, and 0.732, respectively (Fig. 5A). The results of the two validation cohorts, GSE54467 (Fig. 5B, *p* = 0.046, 1-year AUC = 0.432, 3-year AUC = 0.676, 5-year AUC = 0.658) and GSE65904 (Fig. 5C, *p* = 0.003, 1-year AUC = 0.626, 3-year AUC = 0.638, 5-year AUC = 0.570), suggested that a low-risk score patients would have longer disease-free survival (DFS). Age (*p* < 0.05, HR = 1.011, 95% CI [1.001–1.023]), T (*p* < 0.001, HR = 1.380, 95% CI [1.183–1.651]), N (*p* < 0.001, HR = 1.570, 95% CI [1.243–1.984]), and risk score (*p* < 0.001, HR = 1.209, 95% CI [1.091–1.340]) remained significant after multivariate analysis, and these four factors were included in the nomogram model (Fig. 5D). And Fig. 5E showed a calibration curve which suggested the nomogram could predict OS well based on the closeness of anticipated and observed OS values.

### Immune cell infiltration, TME and TMB between the high- and low- risk groups

**Fig. 6:**
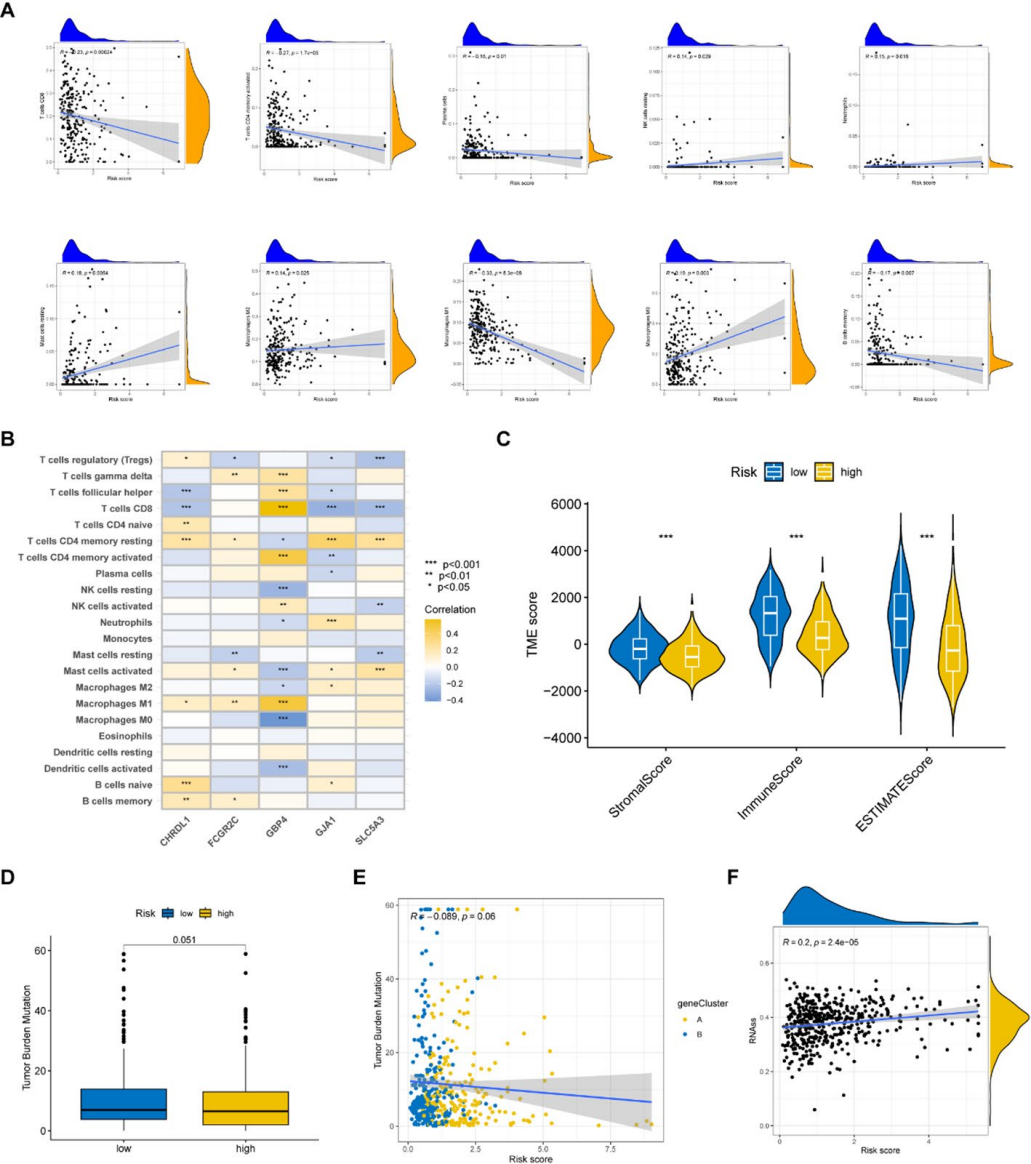
Comparison of tumor characteristics. **A** The correlation between some immune cell types and the risk scores. **B** The correlation between the abundance of immune cells and the five signature genes. **C** The stromal, immunoscore and ESTIMATE scores were compared between the high- and low- risk group.(**D**, **E** The correlation between TMB and risk scores. **F** The correlation between RNA stemness scores (RNAss) and risk scores

We found natural killer (NK) cells resting, neutrophils, mast cells resting, macrophages M2, and macrophages M0 were positively correlated with risk scores, while CD8 T cells, activated memory CD4 T cells, plasma cells, macrophages M1, and memory B cells were negatively correlated with risk scores (Fig. 6A). Figure 6B showed that the five signature genes are correlated with many immune cell infiltrations. Figure 6C demonstrated the low-risk group had a higher stromal score and immune score, which indicated the low-risk group had more immune cell infiltration. Patients in the low-risk group had a higher TMB (Fig. 6D), and the TMB was negatively correlated with risk scores (Fig. 6E), suggesting that the immunotherapy effect may be better in the low-risk group. We also found increased RNA stemness scores (RNAss) were linked to higher risk scores (Fig. 6F).

### Immunotherapy efficacy was evaluated in the high- and low- risk groups

**Fig. 7:**
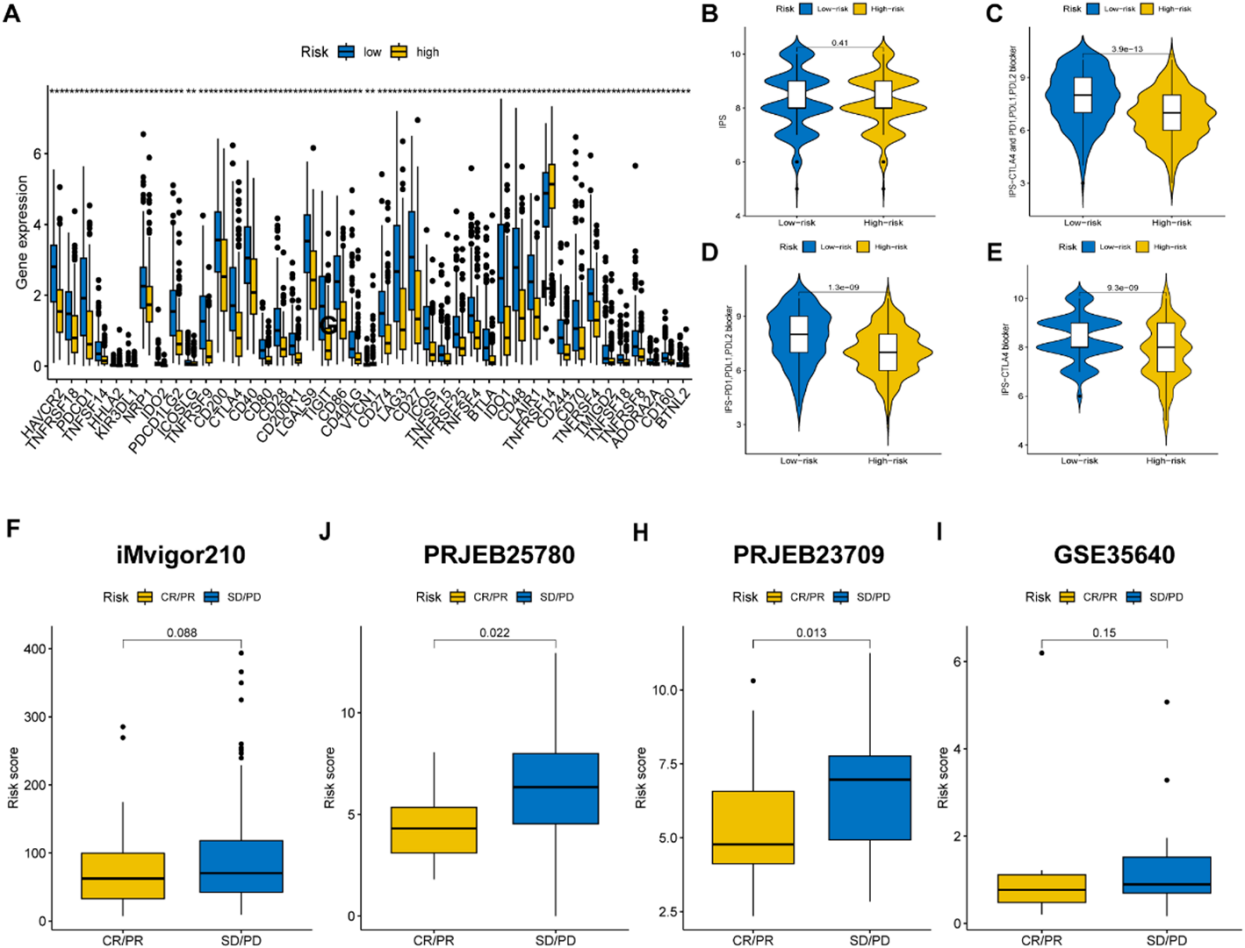
The evaluation of immunotherapy for tumor patients. **A** The differentially expressed immune checkpoint genes were compared between the high- and low-risk groups. **B**–**E** The comparison of immunophenoscores (IPSs) between the two risk groups. **F**–**I** The risk scores were compared between the complete response (CR) / partial response (PR) patients and stable disease (SD)/progressive disease (PD) patients from the iMvigor210, PRJEB25780, PRJNB23709 and GSE35640 cohorts

The low-risk group exhibited elevated expression levels of immune checkpoint genes (Fig. 7A, *p* < 0.05), indicating a potential for improved immunotherapy response. Furthermore, we also found the low-risk group who received different forms of immune checkpoint blockade therapy had significantly elevated immunophenoscores (IPSs) (Fig. 7B–E, *p* < 0.05), suggesting a better response to immunotherapy. We collected four immunotherapy cohorts for evaluating the immunotherapy effect of tumor patients with different risk scores. The tumor patients who had a lower risk showed a higher proportion of responders (Fig. 7F–I). The results indicate the lower risk group would have better responses to immunotherapy.

### Identification of Pathological Features Associated with MRGs

**Fig. 8:**
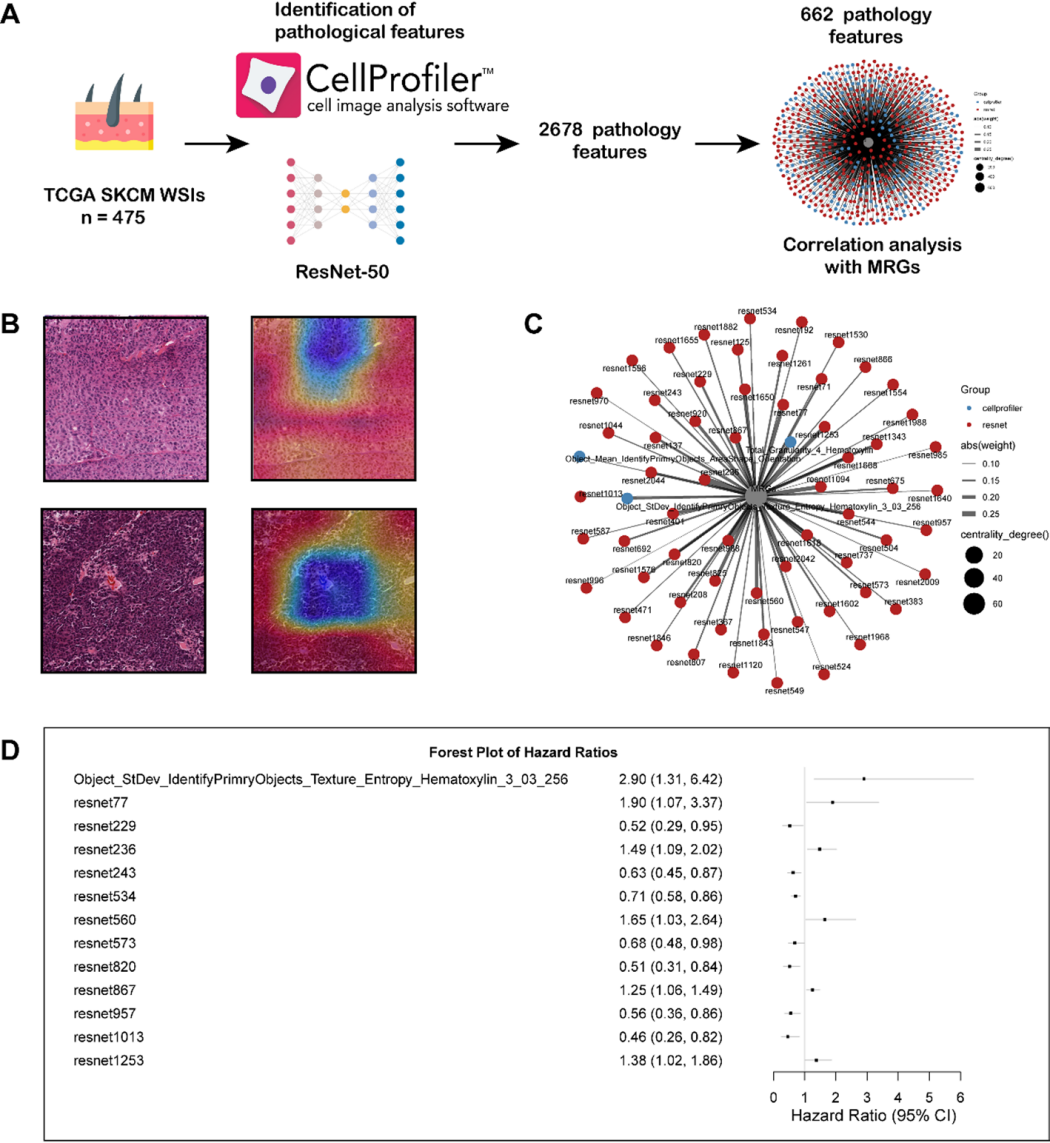
Pathological section analysis identifies pathological features associated with MRGs and prognosis. **A** Flowchart for pathological section analysis of the TCGA-SCKM cohort. **B** The heatmap shows the visualization analysis results of single sample segmentation tiles in the ResNet50 model based on the Grad-CAM algorithm. **C** The network diagram shows 64 pathological features related to MRGs, with red representing those from the ResNet-50 model and blue representing those identified by the cell profiler software. **D** The forest plot illustrates 13 pathological features that demonstrated significant prognostic value in univariate regression analysis

To further expand the clinical application of the MRGs prognostic model, we obtained 475 whole-slide images (WSIs) from the SKCM cohort in TCGA for pathological feature extraction. The workflow is shown in Fig. 8A. After excluding three low-quality slides, we extracted 2,678 pathological features using the ResNet-50 deep learning framework and CellProfiler software, among which 602 features were related to MRGs. The heatmap shown in Fig. 8B visualizes the feature activation distribution of the ResNet-50 model on individual pathological slide tiles using Grad-CAM visualization technology. Considering that multiple features may exhibit collinearity, we further screened the features using elastic regression, obtaining 64 features (Fig. 8C). Through univariate regression analysis, we confirmed 13 pathological features with prognostic significance (Fig. 8D). These analyses further reveal the pathological characteristics associated with MRGs, providing possibilities for the clinical translation of prognostic models.

### scRNA-seq analysis to identify potential communication roles of SLC5A3 + melanoma cells

**Fig. 9:**
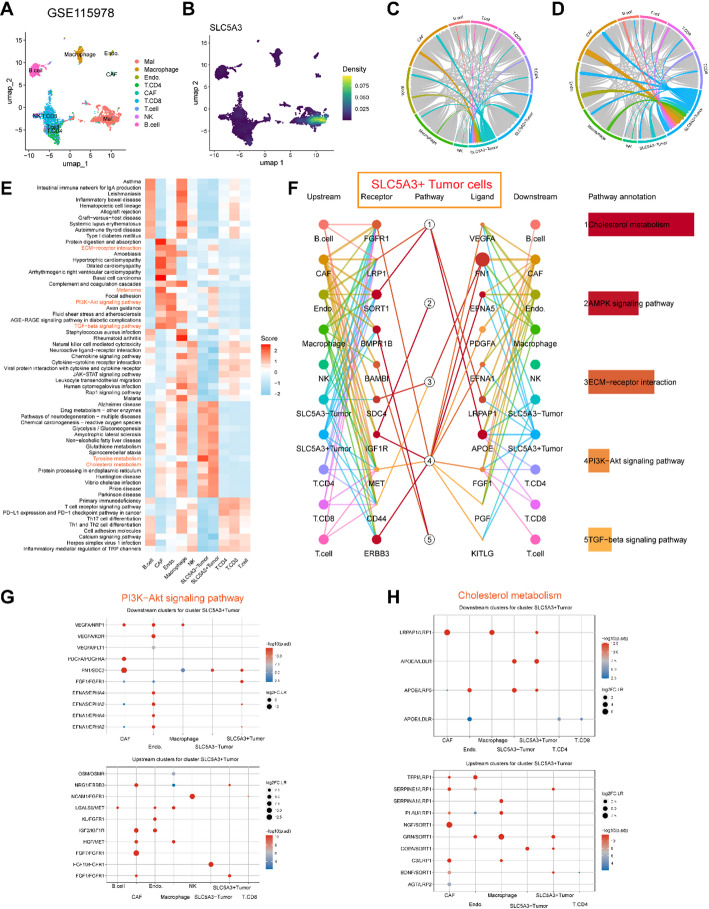
Single-cell transcriptome analysis reveals the role of SLC5A3 in intercellular communication. **A** UMAP visualization of single-cell RNA sequencing data from GSE115978 dataset, showing distinct cell clusters colored by cell type identity. **B** Expression levels of SLC5A3 across different cell clusters, with higher expression levels indicated by darker yellow-green coloration. **C**, **D** Chord diagrams illustrate the intercellular communication network, demonstrating the strength and direction of interactions between SLC5A3 - and SLC5A3 + malignant cells and cell populations in the microenvironment. **E** Heatmap demonstrating differentially expressed pathways in different cell types, with red color indicating up-regulation and blue color indicating down-regulation. **F** Network diagram of SLC5A3 + tumor cells and their intercellular communication pathways constructed using CommPath analysis. The figure depicts five significantly up-regulated pathways and associated receptor-ligand pair interactions in SLC5A3 + tumor cells. The dot plot shows receptor-ligand pairs involving SLC5A3 + tumor cells, which interact with downstream and upstream cell subpopulations in SKCM and are associated with PI3K-AKT pathway activation (**G**) and cholesterol metabolism (**H**)

To explore in which cells these signature genes are primarily expressed in melanoma samples, we performed a single cell sequencing analysis with reference to some public databases. Figure S1A/H and B/I demonstrated 8 kinds of cell types were annotated as the main labels of the SKCM patients and the proportion of each cell type of the patients from cohorts GSE72056 and GSE123139. The FCGR2C and GBP4 majorly express in the immune cells (Figure S1C/J and D/K). The CHRDL1 and GJA1 majorly expressing in the fibroblasts (Figure S1E/L and G/N). The SLC5A3 majorly expresses in the malignant cells (Figure S1F/M). These results demonstrate that the signature genes are expressed in the TME and malignant cells of melanoma tissues. To investigate the function of SLC5A3 in the tumor microenvironment, we performed single-cell RNA sequencing analysis based on the GSE115978 dataset. UMAP dimensionality reduction analysis revealed the presence of multiple distinct cell clusters within the tumor ecosystem, which were annotated as malignant cells, macrophages, tumor-associated fibroblasts (CAFs), CD8 T cells, CD4 T cells, NK cells, endothelial cells, and T cells (Fig. 9A). Detection of SLC5A3 expression in these cell populations revealed heterogeneous expression, with significantly higher expression in malignant cells (Fig. 9B). Based on the median expression of SLC5A3 in malignant cells, we classified them into SLC5A3 + tumor cells and SLC5A3- tumor cells. We found that compared to SLC5A3- tumor cells, SLC5A3 + tumor cells exhibited stronger interactions with CAFs, endothelial cells, and others (Fig. 9C, D). Further pathway enrichment analysis revealed that SLC5A3 + tumor cells exhibit higher activity in pathways related to cholesterol metabolism, the PI3K-Akt signaling pathway, and the TGF-β signaling pathway (Fig. 9E). CommPath analysis also revealed cell-cell receptor-ligand interactions associated with these pathways (Fig. 9F), particularly in the cholesterol metabolism pathway (Fig. 9G) and the PI3K-Akt signaling pathway (Fig. 9H). We found that in both metabolism-related pathways, SLC5A3 + tumor cells exhibit significant communication with CAFs, particularly through the FN1/SDC2, FGF7/FGFR1, NGF/SORT1, and LRPAP1/LRP1 receptor-ligand pairs. This provides additional insight into the potential mechanisms underlying the oncogenic effects of SLC5A3 in tumor cells.

### Validation of the five signature gene expression levels in vitro experiments

**Fig. 10:**
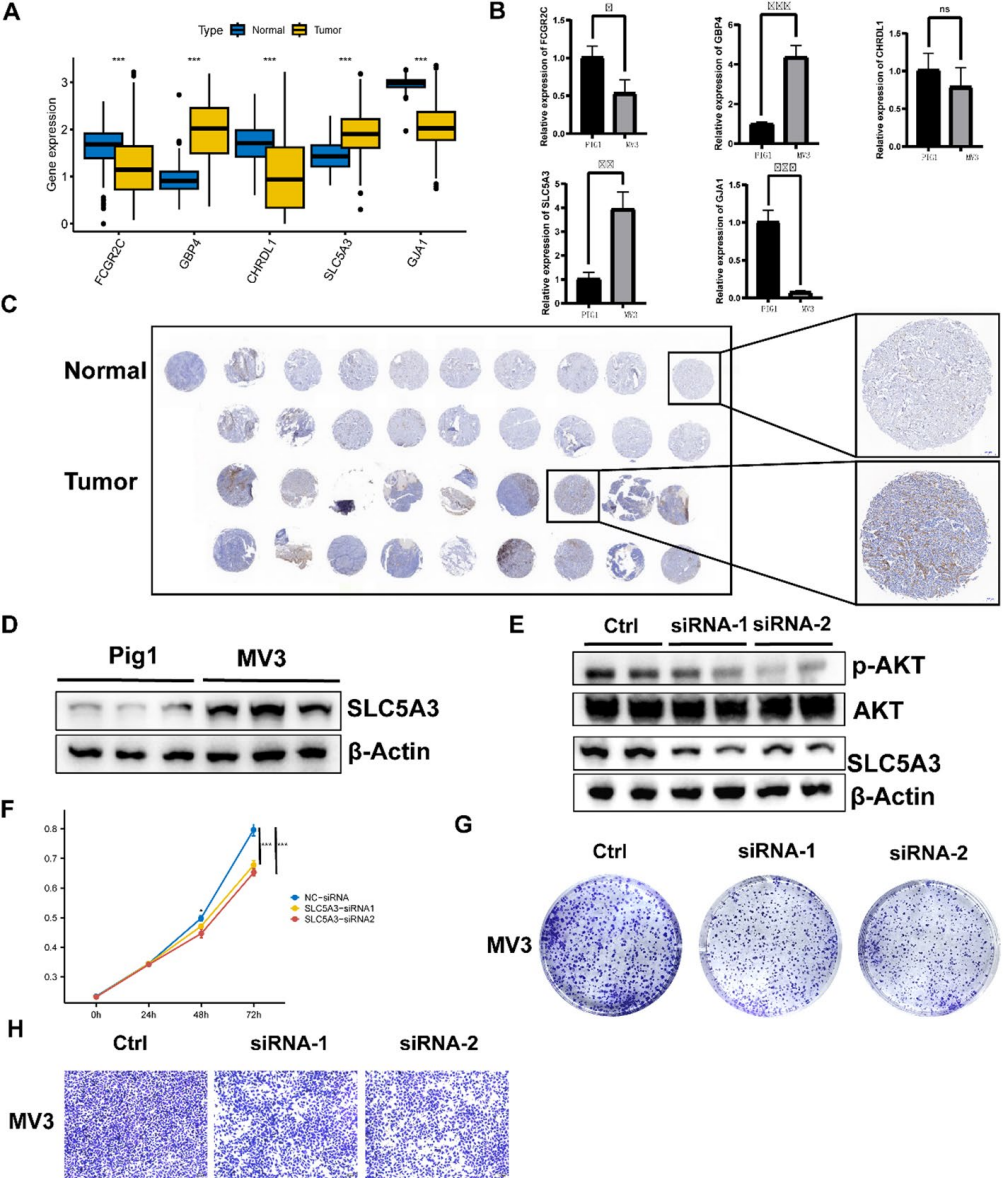
The expression levels of the five signature genes. **A** The comparison of the five signature genes expressions between the normal and tumor tissues. **B** The q-PCR results of the signature gene expression levels between normal melanocytes (PIG1) and SKCM cell lines (MV3). **C** IHC staining results showing SLC5A3 expression levels in melanoma and adjacent normal skin tissue (clinical tissue microarray of melanoma). Scale bar = 200 μm. **D** The WB results of SLC5A3 between normal melanocytes (PIG1) and SKCM cell lines (MV3). **E** The WB results of SLC5A3, AKT and p-AKT in SKCM cell lines (MV3) when silencing SLC5A3 by siRNA. **F**, **G** The CCK8 and colony formation assay results to observe the proliferation of SKCM cell lines (MV3) when silencing SLC5A3 by siRNA. **H** The transwell assay results to observe the migration of SKCM cell lines (MV3) when silencing SLC5A3 by siRNA. *p < 0.05; **p < 0.01; ***p < 0.001

To verify the efficacy of the signature genes we identified in predicting prognosis of SKCM, we compared the expression of each gene between melanoma cells and the healthy controls. We found that GBP4 and SLC5A3 were expressed higher in SKCM patients compared with normal controls, while FCGR2C, CHRDL1 and GJA1 were expressed lower (Fig. 10A). The results of qPCR showed GBP4 and SLC5A3 were higher expressed in SKCM cells (MV3) compared with normal melanocytes (PIG1), while FCGR2C and GJA1 were lower expressed (Fig. 10B), which was in line with our expectations. The results of immunohistochemical (IHC) showed the expression levels of SLC5A3 in SKCM tissues were higher than that in melanin nevus tissue (Fig. 10C). And the results of Western blot (WB) showed the expression levels of SLC5A3 in SKCM cell lines (MV3) were higher than that in melanocytes (PIG1) (Fig. 10D). The above results may indicate that the expression of the signature gene SLC5A3 may promote the development of SKCM.

### Silencing of SLC5A3 inhibits cell proliferation and migration in MV3 cell line

The results of WB showed the SLC5A3 gene of SKCM cells (MV3) was silenced by siRNA and the tumor cells whose SLC5A3 gene was silenced had lower p-AKT expression levels (Fig. 10E). The results of CCK8 assays demonstrated the silence of SLC5A3 could inhibit the proliferation of SKCM cells (Fig. 10F). In addition, in vitro suppression of SLC5A3 inhibits the formation of clones (Fig. 10G). This is consistent with previous findings that reducing p-AKT expression levels inhibits melanoma cell proliferation [15, 16]. Finally, we also found silencing the SLC5A3 gene could impair MV3 cells migration (Fig. 10H).

## Discussion

Over the past few decades, there has been a rise in the occurrence of SKCM. Although the treatment of advanced melanoma has made some progress in recent years, including targeted therapy and immunotherapy, there will still be a large number of advanced melanoma patients will relapse after initial treatment, greatly worsening the prognosis of melanoma patients [16, 17]. Genetic susceptibility also contributes significantly to the development of SKCM, alongside exposure to UV radiation [18]. Genomic research about SKCM has grown rapidly because of a strong association between family history and the disease [19]. The utilization of bioinformatic analysis on genomic data has been extensively employed in constructing prognostic models for SKCM and assessing the risk for patients [20]. In this study, we built a prognostic model of SKCM based on the whole metabolic-related genes (MRGs) that have been proven to be closely related to tumors, which is expected to provide more information for mechanism research and personalized therapy of the disease.

Numerous new biomarkers and potential targets for cancer treatment have been discovered based on altering metabolic genes and metabolites, which has aroused interest in cancer metabolism for an extended period [21, 22]. Many proto-oncogenes and tumor suppressor genes are also implicated in modifying the metabolic pathways of tumors in vivo to control the development and progression of tumors [23]. Our present study has demonstrated that the expression of many MRGs is changed in SKCM patients, which indicates that the expression of MRGs may be associated with the pathogenesis of the disease. Subsequently, we divided the patients into three molecular clusters according to the whole MRGs expression levels. Differences in survival, immune cell infiltration, and immune checkpoint genes expression among the three MRGs molecular clusters indicated the accuracy of the clustering. For the first time, we have linked MRGs to melanoma. Based on these findings, we identified five prognostic signature genes for SKCM among the DEGs among the three molecular subgroups, namely FCGR2, GBP4, CHRDL1, SLC5A3 and GJA1. The expression level of guanylate-binding protein 4 gene (GBP4) has been shown in a previous study increased in melanoma tissue, which is consistent with our experiment [24]. Gap junction protein alpha 1 gene (GJA1), also called connexin 43 (Cx43), has a tumor suppressor function and can inhibit the progression of melanoma [25]. Our research also proved that patients with SKCM exhibit a decreased level of GJA1 expression, potentially contributing to the unfavorable prognosis. We were excited to find for the first time that FCGR2, CHRDL1, SLC5A3 are associated with the prognosis of melanoma. After we identified the signature genes and their risk coefficient, a risk score for each sample could be calculated and SKCM patients could be divided into high- and low- risk groups depending on their respective risk scores.

In recent years, the rise of immunotherapy has greatly controlled the activation of cancer cells and improved the prognosis of many cancer patients [26]. As scientists discovered how to treat cancer by inhibiting negative immune checkpoint regulation, the field was soon applied to melanoma due to its strong immunogenic [27]. ICIs, antibodies that target the cytotoxic T-lymphocyte-associated protein 4 (anti-CTLA-4) and programmed cell death protein 1 (antiPD-1), are used to treat unresectable melanoma, resulting in a dramatic improvement in survival of patients [28]. IPS was usually used to evaluate the immunotherapeutic effect of the ICI [29]. The higher IPS of patients in the low-risk group indicates that they may have a better effect on ICI treatment, which was in line with our expectation. Both TME and tumor-infiltrating immune cells have been identified as important indicators for evaluating tumor immunotherapy and can affect tumor progression [30]. TME includes cancer cells surrounded by various non-malignant cell types and a vascularized extracellular matrix [31]. Activation of CD4 + and CD8 + T cells in adaptive immunity is essential for lifelong remission of melanoma [32]. The exciting thing is that in our study the low-risk group did have more T-cell. We used ESTIMATE algorithm to calculate the proportion of immune-stromal components in TME, which were divided into immune score, stromal score and ESTIMATE score. We found the low-risk group had more immune score, stromal score and ESTIMATE score, which may be because of the more immune cell infiltration. Previous studies have demonstrated that melanoma patients with higher TMB had better effect of ICIs treatment, which is a protective factor for patients’ prognosis [33]. Across the four immunotherapy cohorts, patients of CR /PR had lower risk scores while those of SD /PD had higher risk scores. Overall, patients with a lower risk score will have better prognosis and better response to immunotherapy. Besides of the risk score based on the five signature genes, we also combined clinical features including age, sex, T and N stage to make a nomogram to forecast the prognosis of SKCM patients.

Recently, single-cell RNA-sequencing (scRNA-seq) has emerged in the field of immunology, which can be based on the sequencing of a large number of cells to gain insight into the gene expression of various cell populations in a tissue [34]. We found the SLC5A3 majorly expressed in the malignant cells which suggested it may be correlated with tumor progress. Moreover, we were also surprised to find the expression levels of the five signature genes were significantly different between SKCM patients and healthy controls. The qRT-PCR results showed that the expression levels of four genes, FCGR2, GBP4, SLC5A3 and GJA1, in melanoma cells were different from that in normal melanocyte, which was consistent with the statistical results of the samples we obtained in the public database. We verified the expression of SLC5A3, which had no reported association with tumors, was higher in melanoma tissues than normal tissues using WB and immunohistochemical (IHC) experiment. Furthermore, we also find if SLC5A3 is knocked out, the proliferation capacity of melanoma cells will decrease. Therefore, we verified the accuracy of our MRGs-related prognostic model in predicting the prognosis of SKCM patients from both data analysis and in vivo experiments.

Despite this, our study still has some deficiencies. Although the sample size of the TCGA public database for the identification of signature genes is currently the largest for SKCM patients, a larger sample size is needed to improve statistical efficacy. We lacked tissue samples of clinical cases to confirm our conclusions because SKCM patients are rarely diagnosed in the central part of China.

In conclusion, we established a predictive signature model that can effectively predict prognosis of SKCM patients and assess the responses of patients to immunotherapy. In addition, we identified five signature genes and found that one of their expression, SLC5A3, might promote the proliferation and migration of SKCM cells.

## Methods and materials

### Collection of transcriptional and clinical data

We downloaded the clinical and transcriptome data of 470 SKCM patients from The Cancer Genome Atlas Program (TCGA) database (http://tcga-data.nci.nih.gov/tcga/) (Supplementary Table S2), while the information of 838 healthy controls was acquired from the Genotype-Tissue Expression (GTEx) database. We also collected two SKCM datasets for validation from the Gene Expression Omnibus (GEO) database and the IDs of datasets were GSE54467 [35] and GSE65904 [36] (Supplementary Table S3). In addition, four cohorts of tumor immunotherapy for iMvigor210 (metastatic urothelial carcinoma), PRJEB25780 (metastatic gastric cancers), PRJEB23709 (metastatic melanoma) and GSE35640 [37] (SKCM) were acquired from http://research-pub.gene.com/IMvigor210CoreBiologies, TIDE website (http://tide.dfci.harvard.edu/) and GEO (Supplementary Table S4).

### Genetic alterations of MRGs in SKCM patients

We collected 2752 MRGs from the GO (Gene Ontology) database (Supplementary Table S5). To explore mutations in MRGs in SKCM patients, we analyzed the mutation dataset of the 470 patients from the TCGA database with the R-package maftools [38] and visualized the mutation frequency of MRGs. According to the copy number variation (CNV) data, the frequency of CNV was calculated and the location of CNV occurrence was visualized. Using the R software’s limma package, we utilized the Wilcoxon method to compare the expression levels of MRGs between SKCM patients and healthy controls. The univariate Cox regression method was performed on MRGs based on gene expression data from SKCM patients to evaluate p-values of MRGs.

### Consensus clustering to identify MRGs clusters

To investigate the impact of MRGs on SKCM patients, we conducted consensus clustering analysis on all SKCM samples using expression levels of the 2752 MRGs. Subsequently, we determined the optimal clustering number as K = 3. Principal component analysis (PCA) was employed to distinguish three MRGs groups. To evaluate the value of MRGs, Kaplan-Meier (KM) method and log-rank test with survival and survminer R packages were used to compare overall survival (OS) of the SKCM patients among the three clusters. Additionally, the immune checkpoint genes expression levels were compared among the three MRGs clusters. To investigate the infiltration of immune cells and pathways related to the immune system, we utilized gene set variation analysis (GSVA) and single-sample gene set enrichment analysis (ssGSEA).

### Consensus clustering to identify genes clusters

Consensus clustering analysis was also conducted for all patients based on expression levels of DEGs among the three MRGs clusters, with the optimal clustering number K = 2. The clinical characteristics and MRG expression levels of the two gene clusters were analyzed. Furthermore, we compared the expression levels of MRGs and immune checkpoint genes between the two gene groups. To evaluate the value of DEGs, OS was also compared between the two clusters by the KM method.

### Construction and validation of the DEGs-related prognostic signature

To identify prognostic signature genes, the glmnet R package was utilized to perform LASSO and stepwise Cox regression analyses for all DEGs. Moreover, we constructed a prognostic signature model by using expression levels of the five signature genes and corresponding coefficient values, and then we divided all SKCM patients into low- and high-risk groups according to risk scores. Subsequently, the risk scores were compared among the three MRGs subtypes and the two gene subtypes, respectively. The expression levels of MRGs and five signature genes also were compared between the two different risk groups, respectively. The survival outcomes in different risk groups were analyzed. The KM method was performed to draw the survival curves of the SKCM patients in the two groups from the TCGA database (OS), and the patients from GSE54467 and GSE65904 databases (DFS, disease-free survival) were used for validation. In the meantime, the efficiency of KM curves was evaluated by creating receiver operating characteristic (ROC) curves using the survivalROC *R* package. Moreover, nomogram analysis was performed by combining scoring systems and prediction systems using the *rms* R package. The 1-, 3-, and 5-year survival rates of SKCM patients could be predicted by total points of every factor. The accuracy of the nomogram was demonstrated by using calibration curves.

### Comparison of tumor characteristics between the high- and low- risk groups

The CIBERSORT [39] algorithm was employed to measure the infiltration of immune cells in SKCM tissues, while the Spearman method was utilized to examine the relationship between the risk score and the abundance of infiltrating immune cells. Moreover, we examined the correlation between the immune cells and the five prognostic-related genes. The comparison of TME-related scores of SKCM tissue, including stromal score, immune score, and ESTIMATE score, was conducted between the two risk groups. In addition, TMB was compared between the two risk groups, and the association between TMB and risk scores was also analyzed. At the same time, the linkage between the RNA stemness scores (RNAss) and risk scores was analyzed.

### Comparison of immunotherapy responses between the high- and low-risk groups

To explore the efficacy of the risk scores, we evaluated immunotherapy responses between the two risk groups. Firstly, we examined the expression levels of immune checkpoint genes and the immunophenoscores (IPSs) between the two groups. Moreover, we compared the risk scores between the complete response (CR) / partial response (PR) patients and stable disease (SD) / progressive disease (PD) patients of the four immunotherapy cohorts: iMvigor210, PRJEB25780, PRJEB23709, and GSE35640.

### Pathological feature identification and analysis

Pathological feature extraction and quantitative analysis were performed using a hybrid method combining CellProfiler image analysis software with ResNet50 deep learning architecture. Whole-slide images (WSIs) of 475 SKCM tissue specimens were obtained from the TCGA database. Image preprocessing and slice extraction were performed using the OpenSlide [40] library, generating non-overlapping 512 × 512-pixel slices from the effective tumor region at a magnification of 20× or 10×, excluding areas containing artifacts or excessive background. Comprehensive morphological feature extraction was performed using CellProfiler 4.28 software. For deep learning-based feature extraction, a pre-trained ResNet50 convolutional neural network [41] is used to extract high-dimensional feature representations from tissue pathology sections. Features extracted from the penultimate layer (2048-dimensional vectors) are used as input for downstream prognostic modeling.

### Single-cell sequencing analysis

The expression of the five signature genes in immune and nonimmune cells was analyzed using single-cell sequencing data from two SKCM single-cell datasets (GSE72056 [34] and GSE123139 [42]) from the tumor immune single-cell hub (TISCH) database [43]. Single cell transcriptome data GSE115978 [44] was obtained from GEO database. Comprehensive analysis of single-cell transcriptome data was performed using the Seurat [45] package following established methods from the previous literature [46]. Batch effect correction was subsequently performed using the Harmony package [47]. Cell annotation information was derived from the original literature associated with the dataset, which provided default annotations. Pathway enrichment and cellular communication analysis were performed using the CommPath package to identify dysregulated signaling pathways, catalog ligands and receptors, and characterize potential ligand-receptor interactions [48].

### Validation of gene expression levels in vitro experiment

From the American Type Culture Center (ATCC), a normal human skin melanocyte line (PIG1) and a human melanoma cell line (MV3) were obtained. Cells were cultured at a temperature of 37 °C with 5% CO2 in DMEM (HyClone) supplemented with 10% FBS (Lonsera) and 1% antibiotics (streptomycin and penicillin).

### RNA isolation and quantitative PCR

RNA was isolated from cell lines using TRIzol kit (Carlsbad), and its concentration was determined using a UV spectrophotometry (ThermoFisher Scientific). Next, the mRNA was reverse transcribed into cDNA using PrimeScript RT Master Mix (TaKaRa). The quantitative detection of cDNA was performed using SYBR Green Premix Pro Taq HS qPCR Kit. Finally, the 2^−∆∆Ct^ method was utilized to calculate the relative mRNA expression. The primer sequences of the signature genes for qPCR are shown in Supplementary Table S6.

### Western blot

To extract total proteins from cells, RIPA buffer (Beyotime, China) was used along with the addition of protease inhibitor and phosphatase inhibitor. Then the proteins were run on a 10% SDS-PAGE gel and transferred onto nitrocellulose membranes for immunoblotting. The primary antibodies used in this study were as follows: β-actin (Cell Signaling Technology), SLC5A3 (Leading Biology), p-AKT (Cell Signaling Technology), AKT (Cell Signaling Technology).

### Melanoma tissue microarray and immunohistochemical staining

The human melanoma tissue and normal skin tissue microarray (Cat No. ZL-MEL361) was obtained from ShangHai Zhuoli Biotech Company (China), including 9 melanoma tissue samples and 9 normal skin tissue samples. ShangHai Zhuoli Biotech Company Ethics Committee approved the experiments (Ethics Approval Number: SHLLS-BA-22101102). IHC staining was carried out to measure SLC5A3 protein levels in melanoma tissues per the manufacturer’s instructions using an anti-SLC5A3 antibody (1:500, Proteintech).

### Cell proliferation and migration after Silencing SLC5A3 expression

SLC5A3 expression was silenced in MV3 cells using siRNA (Tsingke Biotechnology, 1 × 10^5 cells/µl) with the transfection reagent Lipo3000 (Thermo, 1 × 10^5 cells/µl), and the two sequences used were displayed in Supplementary Table S7.

### CCK8 methods

We cultured MV3 cells (200000/well) whose SLC5A3 were silenced. After culturing these cells for 0, 24, 48, or 72 h, we added the CCK8 solution (Cell Counting Kit-8, Beyotime) and continued culturing for 1.5 h. An optical density (OD) value at 450 nm was observed for assessing the proliferation ability.

### Colony formation assay

To examine the effects of SLC5A3 expression on SKCM cells proliferation, we cultured MV3 cells (1500/well) whose SLC5A3 were silenced. After 12 days, the number of colonies were counted.

### Transwell experiment

The transwell assay was performed using transwell chambers (Corning, NY, USA). MV3 cells transfected with siRNA were suspended in 200 µl of serum-free medium and placed in the upper chamber of the transwell, while a medium containing 10% fetal bovine serum was added to the lower chambers. Following a 36-hour incubation period, the inner surface of the chambers was gently scrubbed, and the cells on the opposite side of the membrane were fixed with a 4% formaldehyde solution, stained with crystal violet, and observed under a microscope.

### Statistical analysis

All experiments were performed in accordance with relevant guidelines and regulations. Each experiment in this study was replicated independently a minimum of three times. Data analysis was performed using R4.3.1, a significance level of *P* < 0.05 was deemed statistically significant.

## Electronic supplementary material

Below is the link to the electronic supplementary material.

Supplementary Material 1: Table S1: Clinical information of 470 melanoma cancer patients (TCGA). Table S2: Clinical information of validation cohorts (GSE65904; GSE54467). Table S3: Clinical information of immunotherapy cohorts (iMvigor210; GSE35640; PRJEB25780; PRJEB23709). Table S4: Summary of 2752 metabolic-related genes. Table S5: The primer sequences for qRT-PCR. Table S6: Coefficient values of 5 signature genes in the multivariate Cox analysis. Table S7: The primer sequences of siRNA for silencing the SLC5A3 gene.

Supplementary Material 1: Figure S1: Cellular landscape of the tumor microenvironment in SKCM revealed by single-cell transcriptomic analysis of TISCH cohorts.

## Data Availability

Gene expression and clinical data included in this study was obtained from the following publicly available datasets: The Cancer Genome Atlas Program (TCGA) database (http://tcga-data.nci.nih.gov/tcga/), Genotype-Tissue Expression (GTEx) database Genotype-Tissue Expression (GTEx) database (https://commonfund.nih.gov/GTEx), Gene Expression Omnibus (GEO) database (http://www.ncbi.nlm.nih.gov/geo), IMvigor210 dataset (http://research-pub.gene.com/IMvigor210CoreBiologies) and TIDE website (http://tide.dfci.harvard.edu/).
