# Supplementary material for: Development and validation of a prognostic model for predicting survival and immunotherapy benefits in melanoma based on metabolism-relevant genes
Source: Discov Oncol. 2025 Jul 12;16:1321. doi: 10.1007/s12672-025-03186-8 (PMC12255649; doi:10.1007/s12672-025-03186-8)
Supplement: Supplementary file 2 — Supplementary Material 1: Figure S1: Cellular landscape of the tumor microenvironment in SKCM revealed by single-cell transcriptomic analysis of TISCH cohorts. [file 12672_2025_3186_MOESM2_ESM.docx]

**
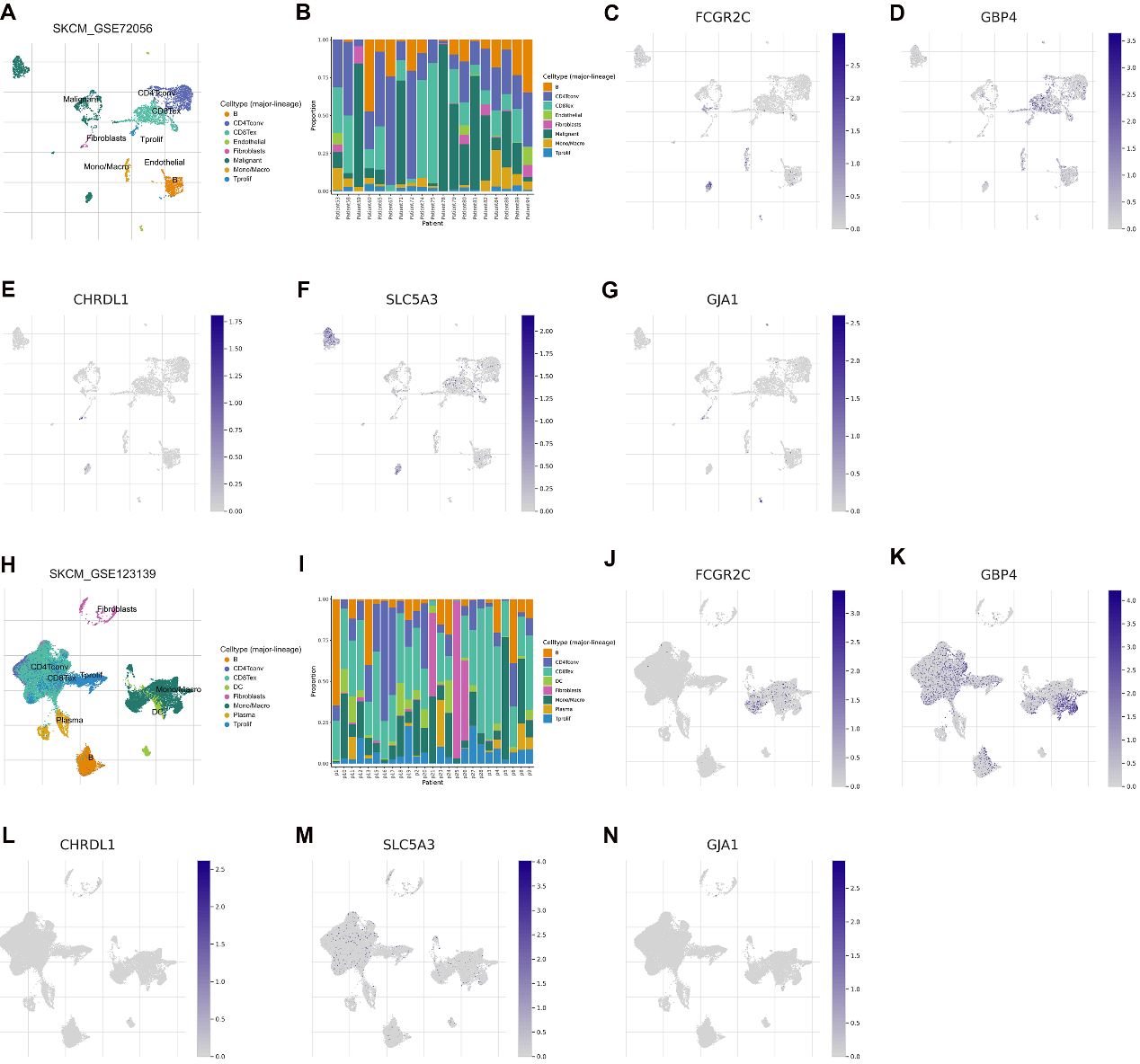
**

**Figure S1. Profiles of cell types in TME of SKCM on the scRNA transcriptome level.** (A) 8 kinds of cell types were annotated as the main labels of the SKCM patients from cohort GSE72056. (B/I) The proportion of each cell type of the patients. (C-G and J-N) The distribution of the five signature genes expression. (H) 8 kinds of cell types were annotated as the main labels of the SKCM patients from cohort GSE123139.
